# Supplementary material for: Impact of Interobserver Variability in Manual Segmentation of Non-Small Cell Lung Cancer (NSCLC) Applying Low-Rank Radiomic Representation on Computed Tomography
Source: Cancers (Basel). 2021 Nov 28;13(23):5985. doi: 10.3390/cancers13235985 (PMC8657389; doi:10.3390/cancers13235985)
Supplement: Supplementary file 1 [file cancers-13-05985-s001.zip › cancers-1362609-supplementary.pdf]

Supplementary Materials: Impact of Interobserver Variability in Manual Segmentation of Non-Small Cell Lung Cancer (NSCLC) Applying Low-Rank Radiomic Representation on Computed Tomography

Michelle Hershman, Bardia Yousefi, Lacey Serletti, Maya Galperin-Aizenberg, Leonid Roshkovan, José Marcio Luna, Jeffrey C. Thompson, Charu Aggarwal, Erica L. Carpenter, Despina Kontos and Sharyn I. Katz

Supplementary Materials: The following are available online at www.mdpi.com/xxx/s1, Supplement Table S1: The CT parameters for Stanford NSCLC Radiogenomics dataset, Supplement Table S2 The CT parameters for Harvard NSCLC Radiomics-Genomics dataset, Supplement Table S3: Similarity of the radiomic signatures using Pearson correlation among different segmenters are presented for different stratifications based on CT parameters, Supplement Table S4: Overall survival, Cox regression. Using the low-rank representation of the radiomic signatures survival prediction is measured for each segmenter while there is Contrast-Enhancement (CE), Supplement Table S5: Overall survival, Cox regression. Using the low-rank representation of the radiomic signatures survival prediction is measured for each segmenter while there is Non-Contrast-Enhanced (UN), Supplement Table S6: Overall survival, Cox regression. Using the low-rank representation of the radiomic signatures survival prediction is measured for each segmenter for higher convolutional kernel (CKh). Supplement Table S7: Overall survival, Cox regression. Using the low-rank representation of the radiomic signatures survival prediction is measured for each segmenter for slice thickness between 2mm and 4mm. Table S8. Itraclass correlation coefficient based on radiomics categories and with the respect of different group means. For each segmenter, mean and standard deviation of correlation coefficient is calculated for every radiomics' category, Table S9. Radiomic features with lesser stability with the respect to different segmenters. Means and standard deviations of these radiomics are presented. Table S10. More detailed information about the Radiomic features used in this study., Table S11. The hazard ratio for each covariate in the maximal cox proportional hazard model.

Table S1. The CT parameters for Stanford NSCLC Radiogenomics dataset.

	**CT Imaging Parameters**			
**Characteristic**	**Siemens Healthineers**	**Ge Healthcare**	**Philips Medical Systems Tech.**	**Toshiba Corporation**
**Contrast Enhanced**	24	71	2	-
**Non- Contrast Enhanced**	34	71	4	1
**Convolution Kernel**				
**Standard (soft-tissue)**	4	43	3 (2(YB), 1(B))	-
**Hard (lung, bone, boneplus)**	54	99	3(C)	1 (FC52)
**Exposure (*mAs*)**	159.5 (30, 466)	447.5 (31.8,754)	217 (93, 352)	217
**Focal Spots (*mm*)**				
**0.8**	1	14	-	-
**1.2**	57	118	-	1
**Unknown**	-	10	6	-
**Tube Voltage (*kVp*)**	120 (100,130)	120 (100,140)	unknown	unknown
**Reconstruction Diameter (*mm*)**	358.5 (235,500)	400 (302,500)	350 (304,384)	474.6
**Slice Thickness *(mm)***				
≤1	49	14	3	-
1< .≤2	4	100	-	1
2< ≤4	4	27	2	-
≤5	1	1	1	-

Table S2. The CT parameters for Harvard NSCLC Radiomics-Genomics dataset.

	**CT imaging parameters**		
**Characteristic**	**Siemens Healthineers**	**Ge Healthcare**	**Philips Medical Systems Tech.**

**Contrast Enhanced**	26	1	25
**Non- Contrast Enhanced**	12	-	23
**Convolution Kernel**			
**Standard (soft-tissue)**	3	-	40 (30:B,10:A)
**Hard (lung, bone,boneplus)**	35	1	8 (1:C, 7:D)
			-
**Exposure (*mAs*)**	125 (58, 158)	5	119 (20, 390)
**Focal Spots (*mm*)**			
**0.95**	6	1	-
**1.2**	32	-	-
**Unknown**	-	-	48
**Tube Voltage (*kVp*)**	120 (120,140)	120	-
**Reconstruction Diameter (*mm*)**	465 (308, 700)	402	430 (311, 600)
**Slice Thickness *(mm)***			
≤1	-	-	-
1<..≤2	-	-	4
2< ≤4	7	1	17
≤5	31	-	27

Table S3. Similarity of the radiomic signatures using Pearson correlation among different segmenters are presented for different stratifications based on CT.

**NSCLC Dataset**	**Segmenters ID**	**Similarity Among Segmentations**			
		**Correlation Score**			
		**CE**	**UN**	**CKh**	**ST**
**LUNG3 NSCLC-Radiomics-Genomics Harvard Dataset**	**BY**	0.95	0.79	0.84	0.96
	**LS**	0.92	0.93	0.96	0.97
	**MH**	0.89	0.97	0.93	0.97
**NSCLC-Radiogenomics Stanford Dataset**	**BY**	0.92	0.96	0.87	0.91
	**LS**	0.86	0.97	0.78	0.91
	**MH**	0.74	0.89	0.53	0.90

CE-Contrast-enhanced; UN-unenhanced; CKh-convolutional kernel (hard); ST- slice thickness parameters.

Table S4. Overall survival, Cox regression. Using the low-rank representation of the radiomic signatures survival prediction is measured for each segmenter while there is Contrast-Enhancement (CE).

**NSCLC Datasets**	**Modeling covariates**	**Prediction Survival**							
		**BY**		**LS**		**MH**		**SK-GT**	
		**c-statistic (95% CI)**	***p* versus null[1]**	**c-statistic (95% CI)**	***p* versus null[1]**	c-statistic (95% CI)	***p* versus null[1]**	**c-statistic** (95% CI)	***p* versus null[1]**
**LUNG3 NSCLC-Radiomics-Genomics Harvard Dataset CE**	clinical & demographic[2]							0.71	0.02
	Three PC radiomic signatures	0.66	0.06	0.67	0.04	0.63	0.3	0.7	0.03
	Radiomic signatures, clinical & demographic	0.73	0.2	0.71	0.2	0.7	0.3	0.72	0.2
**NSCLC-Radiogenomics**	clinical & demographic[3]							0.71	0.02

**Stanford Dataset CE**	Three PC radiomic signatures	0.69	0.03	0.65	0.02	0.61	0.3	0.68	0.01
	Radiomic signatures, clinical & demographic	0.77	0.002	0.75	0.001	0.75	0.005	0.76	0.001

CI: confidence interval. 1. p value by likelihood ratio test versus the hypothesis that the model is no better than the null model. 2. Clinical and demographic covariates for LUNG3-NSCLC-Radiomics-Genomics Harvard Dataset: sex, stage status, and histology. 3. Clinical and demographic covariates for NSCLC-Radiogenomics Stanford Dataset: sex, stage status, and histology.

Table S5. Overall survival, Cox regression. Using the low-rank representation of the radiomic signatures survival prediction is measured for each segmenter while there is Non-Contrast-Enhanced (UN).

		**Prediction survival**							
		**BY**		**LS**		**MH**		**SK-GT**	
**NSCLC Datasets**	**Modeling covariates**	**c-statistic (95% CI)**	***p* versus null[1]**	**c-statistic (95% CI)**	***p* versus null[1]**	**c-statistic** (95% CI)	***p* versus null[1]**	**c-statistic** (95% CI)	***p* versus null[1]**
**LUNG3 NSCLC-Radiomics-Genomics Harvard Dataset UN**	clinical & demographic[2]							0.71	0.02
	Three PC radiomic signatures	0.69	0.03	0.65	0.02	0.61	0.3	0.68	0.01
	Radiomic signatures, clinical & demographic	0.77	0.002	0.75	0.001	0.75	0.005	0.76	0.001
**NSCLC-Radiogenomics Stanford Dataset UN**	clinical & demographic[3]							0.66	0.08
	Three PC radiomic signatures	0.63	0.05	0.67	0.05	0.67	0.08	0.6	0.2
	Radiomic signatures, clinical & demographic	0.64	0.2	0.68	0.05	0.69	0.3	0.65	0.3

CI: confidence interval. 1. p value by likelihood ratio test versus the hypothesis that the model is no better than the null model. 2. Clinical and demographic covariates for LUNG3-NSCLC-Radiomics-Genomics Harvard Dataset: sex, stage status, and histology. 3. Clinical and demographic covariates for NSCLC-Radiogenomics Stanford Dataset: sex, stage status, and histology.

Table S6. Overall survival, Cox regression. Using the low-rank representation of the radiomic signatures survival prediction is measured for each segmenter for higher convolutional kernel (CKh).

		**Prediction survival**							
		**BY**		**LS**		**MH**		**SK-GT**	
**NSCLC Datasets**	**Modeling covariates**	**c-statistic (95% CI)**	***p* versus null[1]**	**c-statistic (95% CI)**	***p* versus null[1]**	**c-statistic** (95% CI)	***p* versus null[1]**	**c-statistic** (95% CI)	***p* versus null[1]**
**LUNG3**	clinical & demographic[2]							0.57	0.5

**NSCLC-Radiomics-Genomics Harvard Dataset CKh**	Three PC radiomic signatures	0.62	0.4	0.61	0.4	0.6	0.5	0.7	0.03
	Radiomic signatures, clinical & demographic	0.65	0.4	0.61	0.6	0.64	0.3	0.77	0.02
**NSCLC-Radiogenomics Stanford Dataset CKh**	clinical & demographic[3]							0.7	<0.005
	Three PC radiomic signatures	0.68	<0.005	0.7	0.003	0.67	0.04	0.7	<0.005
	Radiomic signatures, clinical & demographic	0.74	<0.005	0.76	<0.005	0.75	0.002	0.75	<0.005

CI: confidence interval. 1. p value by likelihood ratio test versus the hypothesis that the model is no better than the null model. 2. Clinical and demographic covariates for LUNG3-NSCLC-Radiomics-Genomics Harvard Dataset: sex, stage status, and histology. 3. Clinical and demographic covariates for NSCLC-Radiogenomics Stanford Dataset: sex, stage status, and histology.

Table S7. Overall survival, Cox regression. Using the low-rank representation of the radiomic signatures survival prediction is measured for each segmenter for slice thickness between 2mm and 4mm.

		**Prediction survival**							
		**BY**		**LS**		**MH**		**SK-GT**	
**NSCLC Datasets**	**Modeling covariates**	**c-statistic (95% CI)**	***p* versus null[1]**	**c-statistic (95% CI)**	***p* versus null[1]**	**c-statistic** (95% CI)	***p* versus null[1]**	**c-statistic** (95% CI)	***p* versus null[1]**
**LUNG3 NSCLC-Radiomics-Genomics Harvard Dataset ST**	clinical & demographic[2]							0.7	0.4
	Three PC radiomic signatures	0.69	0.6	0.61	0.8	0.67	0.4	0.64	0.5
	Radiomic signatures, clinical & demographic	0.77	0.5	0.72	0.7	0.78	0.2	0.77	0.4
**NSCLC-Radiogenomics Stanford Dataset ST**	clinical & demographic[3]							0.66	0.5
	Three PC radiomic signatures	0.5	0.9	0.63	0.7	0.58	0.9	0.6	0.7
	Radiomic signatures, clinical & demographic	0.76	0.5	0.65	0.8	0.73	0.7	0.79	0.5

CI: confidence interval. 1. p value by likelihood ratio test versus the hypothesis that the model is no better than the null model. 2. Clinical and demographic covariates for LUNG3-NSCLC-Radiomics-Genomics Harvard Dataset: sex, stage status, and histology. 3. Clinical and demographic covariates for NSCLC-Radiogenomics Stanford Dataset: sex, stage status, and histology.

Table S8. Itraclass correlation coefficient based on radiomics categories and with the respect of different group means. For each segmenter, mean and standard deviation of correlation coefficient is calculated for every radiomics' category.

	**Similarity Among Segmenters for Radiomics Categories**			
	**Radiomics Category**	**Correlation BY**	**Correlation LS**	**Correlation MH**
**LUNG3 NSCLC-Radiomics-Genomics Harvard Dataset**	**Shape-Based**	0.75(±0.15)	0.79(±0.2)	0.82(±0.14)
	**1st-Order**	0.81(±0.24)	0.85(±0.2)	0.84(±0.18)
	**GLCM**	0.71(±0.27)	0.82(±0.1)	0.74(±0.21)
	**GLSM**	0.72(±0.2)	0.7(±0.2)	0.73(±0.2)
	**GLRLM**	0.72(±0.3)	0.79(±0.2)	0.76(±0.2)
	**NGTDM**	0.65(±0.2)	0.71(±0.16)	0.75(±0.1)
	**GLDM**	0.73(±0.3)	0.78(±0.2)	0.77(±0.2)
	**LOG**	0.78(±0.1)	0.8(±0.1)	0.81(±0.2)
	**Wavelet**	0.88(±0.14)	0.86(±0.2)	0.89(±0.17)
**NSCLC**-Radiogenomics **Stanford Dataset**	**Shape-Based**	0.74(±0.13)	0.72(±0.14)	0.74(±0.1)
	**1st-Order**	0.69(±0.2)	0.67(±0.16)	0.7(±0.18)
	**GLCM**	0.73(±0.2)	0.67(±0.1)	0.7(±0.16)
	**GLSM**	0.82(±0.1)	0.7(±0.1)	0.79(±0.1)
	**GLRLM**	0.82(±0.07)	0.8(±0.1)	0.7(±0.06)
	**NGTDM**	0.4(±0.2)	0.48(±0.2)	0.53(±0.3)
	**GLDM**	0.8(±0.1)	0.77(±0.2)	0.76(±0.1)
	**LOG**	0.82(±0.1)	0.75(±0.1)	0.77(±0.2)
	**Wavelet**	0.83(±0.2)	0.8(±0.1)	0.78(±0.2)

Table S9. Radiomic features with lesser stability with the respect to different segmenters. Means and standard deviations of these radiomics are presented.

	**Less Stable Radiomics**	
**Radiomic Types**	**Radiomics name**	**Correlation average**
**Shape-based**	Sphericity	0.51 (±0.02)
	Kurtosis	0.49 (±0.07)
**First-Order statistic**	MeanAbsoluteDeviation	0.6 (±0.08)
	Skewness	0.44 (±0.1)
**GLCM**	ClusterProminence	0.41(±0.4)
	ClusterShade	0.42 (±0.44)

	ClusterTendency	0.54 (±0.25)
	DifferenceVariance	0.67(±0.13)
	SumAverage	0.61 (±0.001)
	SumSquares	0.57 (±0.2)
**GLSM**	LargeAreaLowGrayLevelEmphasis<br>LowGrayLevelZoneEmphasis	0.39(±0.1)<br>0.4(±0.07)
	SmallAreaLowGrayLevelEmphasis	0.47(±0.07)
	GrayLevelVariance	0.58(±0.2)
	HighGrayLevelRunEmphasis	0.63(±0.03)
**GLRLM**	LongRunHighGrayLevelEmphasis	0.59(±0.04)
	LongRunLowGrayLevelEmphasis	0.33(±0.1)
	LowGrayLevelRunEmphasis	0.47(±0.1)
	ShortRunLowGrayLevelEmphasis	0.51(±0.08)
**NGTDM**	Busyness	0.6(±0.2)
	Strength	0.55(±0.1)
	GrayLevelVariance	0.58(±0.2)
**GLDM**	HighGrayLevelEmphasis	0.62(±0.02)
	LargeDependenceLowGrayLevelEmphasis	0.33(±0.1)
	LowGrayLevelEmphasis	0.48(±0.1)
	log-sigma-4-0-mm-3D_RobustMeanAbsoluteDeviation	0.66(±0.02)
	log-sigma-4-0-mm-3D_Variance	0.57(±0.2)
	log-sigma-3-5-mm-3D_Kurtosis	0.69(±0.07)
**LOG**	log-sigma-3-5-mm-3D_RobustMeanAbsoluteDeviation	0.64(±0.02)
	log-sigma-3-5-mm-3D_Variance	0.57(±0.2)
	log-sigma-2-5-mm-3D_Variance	0.61(±0.14)
	log-sigma-2-0-mm-3D_90Percentile	0.48(±0.1)
	log-sigma-1-5-mm-3D_90Percentile	0.34(±0.15)
	log-sigma-1-0-mm-3D_90Percentile	0.3(±0.2)
	wavelet-LHH_Mean	0.53(±0.1)
**Wavelet**	wavelet-HLH_Mean	0.5(±0.2)
	wavelet-HLH_Skewness	0.59(±0.03)
	wavelet-HHL_Mean	0.52(±0.2)
	wavelet-HHL_Median	0.52(±0.2)
	wavelet-HHH_Mean	0.24(±0.2)
	wavelet-HHH_Median	0.23(±0.2)

Table S10. More detailed information about the Radiomic features used in this study.

**Radiomic Features Information**	
**Quantization method**	Fixed-bin width
**Bin-width size**	25
**Median bin counts (range) for original image**	90 (40–140)
**Median bin counts (range) for all the filters**	85 (30–120)
**Laplcian of Gaussian (LoG)**	LoG with 10 sigma levels (0-5mm, strides of 0.5mm)<br>For each level, 18 features from first-order statistics were extracted
**Wavelet**	Three channels<br>For each level, 18 features from first-order statistics were extracted

Table S11. The hazard ratio for each covariate in the maximal cox proportional hazard model.

		Hazard ratio			
	**Covariates added to model**	**BY** ***p*-value**	**LS** ***p*-value**	**MH** ***p*-value**	**SK** ***p*-value**
**LUNG3** NSCLC-Radiomics-Genomics **Harvard Dataset**	**PC1**	1.0 (0.98-1.0) 0.8	1.00 (0.97-1.0) 0.8	0.99 (0.96-1.0) 0.3	1.00 (0.97-1.0) 0.9
	**PC2**	1.02(0.99-1.0) 0.1	1.03 (1.00-1.1) 0.03	1.02 (0.99-1.1) 0.1	1.04 (1.01-1.1) 0.007
	**PC3**	1.0 (0.95-1.0) 0.8	1.04 (1.00-1.1) 0.03	1.02 (0.96-1.1) 0.5	0.96 (0.92–1.0) 0.08
	**Histology**	0.92 (0.61-1.4) 0.7	0.83 (0.55-1.3) 0.3	0.87 (0.58-1.3) 0.5	0.69 (0.43-1.1) 0.1
	**Stage**	1.32 (0.91-1.9) 0.1	1.38 (0.97-2.0) 0.07	1.23 (0.85-1.8) 0.2	1.34 (0.93-1.9) 0.1
	**Gender**	1.32 (0.67-2.6) 0.4	1.36 (0.70-2.7) 0.3	1.3 (0.66-2.6) 0.4	1.32 (0.66-2.6) 0.4
**NSCLC**-Radiogenomics **Stanford Dataset**	**PC1**	1.0 (0.98-1.0) 0.8	1.00 (0.97-1.0) 0.8	1.02 (0.99-1.0) 0.1	1.0 (0.98-1.0) 0.7
	**PC2**	1.06 (1.02-1.1) 0.003	1.03 (1.00-1.1) 0.03	1.03 (1.01-1.1) 0.01	1.1 (1.02-1.1) 0.001
	**PC3**	0.95 (0.90-1.0) 0.1	0.99 (0.95-1.0) 0.7	0.97 (0.92-1.0) 0.3	1.1 (1.00-1.1) 0.06
	**Histology**	0.83 (0.35-1.9) 0.5	0.92 (0.46-1.9) 0.8	0.94 (0.47-1.9) 0.8	1.0 (0.49-2.0) 0.9
	**Morphology**	0.82 (0.35-1.9) 0.6	1.06 (0.47-2.4) 0.8	0.95 (0.41-2.2) 0.9	1.5 (0.69-3.1) 0.3
	**Gender**	3.46 (1.41-8.5) 0.007	3.59 (1.46-8.8) 0.005	3.27 (1.33-8.1) 0.01	3.4 (1.35-8.3) 0.009
